# Quantitative characterization of myocardial infarction by cardiovascular magnetic resonance predicts future cardiovascular events in patients with ischemic cardiomyopathy

**DOI:** 10.1186/1532-429X-10-17

**Published:** 2008-04-09

**Authors:** Hajime Yokota, Shahriar Heidary, Chandra K Katikireddy, Patricia Nguyen, John M Pauly, Michael V McConnell, Phillip C Yang

**Affiliations:** 1 Division of cardiovascular medicine, department of medicine, Stanford university, Stanford, CA, USA; 2 Department of electrical engineering, Stanford university, Stanford, CA, USA

## Abstract

**Background:**

Cardiovascular magnetic resonance (CMR) can provide quantitative data of the myocardial tissue utilizing high spatial and temporal resolution along with exquisite tissue contrast. Previous studies have correlated myocardial scar tissue with the occurrence of ventricular arrhythmia. This study was conducted to evaluate whether characterization of myocardial infarction by CMR can predict cardiovascular events in patients with ischemic cardiomyopathy (ICM).

**Results:**

We consecutively studied 86 patients with ICM (LVEF < 50%, mean LVEF: 26 ± 12%) with CMR before revascularization or medication therapy ± implantable cardiac defibrillator, determined the amount of myocardial scar, and followed for development of cardiovascular events. Thirty-three patients (38%) had cardiovascular events (mean follow-up: 20 ± 16 months). Patients who developed cardiovascular events had larger scar volume and scar percentage of the myocardium than those who did not develop cardiovascular events (16.8 ± 12.4 cm3 vs. 11.7 ± 12.6 cm3, p = 0.023 and 10.2 ± 6.9% vs. 7.2 ± 6.7%, p = 0.037, respectively). There were no significant differences in LVEDV, LVESV and LVEF between the patients with and without cardiovascular events (231 ± 76 ml vs. 230 ± 88 ml; 180 ± 73 ml vs. 175 ± 90 ml; and 25 ± 10% vs. 27 ± 13%, respectively).

**Conclusion:**

Quantification of the scar volume and scar percentage by CMR is superior to LVEDV, LVESV, and LVEF in prognosticating the future likelihood of the development of cardiovascular events in patients with ICM.

## Background

Congestive heart failure (CHF) has become a widespread public health concern, affecting approximately 5 million patients in the United States. Over 550,000 new cases and 300,000 deaths are reported annually [1]. The most common cause of CHF is coronary artery disease. Of these, the highest mortality rate is seen in patients with ischemic cardiomyopathy (ICM) [2]. The high morbidity and mortality in CHF have been associated with a high incidence of ventricular arrhythmia and left ventricular (LV) remodelling [3,4]. Post infarction LV remodelling provides a substrate to trigger high-grade ventricular arrhythmia [5]. Specifically, areas of peri-infarct ischemia have been shown to be arrhythmogenic. It has been well known that revascularization of these ischemic territories results in a lower incidence of ventricular arrhythmia in patients with ICM [6-9]. Similarly, scar tissue has been associated with ventricular arrhythmia [10-12]. Previous studies have reported that myocardial scar as assessed by cardiovascular magnetic resonance (CMR), is more accurate than traditional measurements including left ventricular ejection fraction (LVEF) in identifying patients who develop ventricular arrhythmia [13-17]. However, these studies were not conducted in patients with significantly compromised LV function, who are most predisposed to developing cardiovascular events, including ventricular arrhythmia, pathological remodelling and worsening CHF. Furthermore, prognostic relationship between transmurality of the scar and occurrence of cardiovascular events in patients with ICM was not investigated. In order to address these issues, we systematically analyzed multiple imaging parameters generated from CMR to determine whether the characterization and quantitation of myocardial infarction (MI) by CMR can predict cardiovascular events in patients with ICM.

## Methods

### Patient population

Eighty-six patients (73 men, 13 women; mean age 57 ± 12 years) with coronary artery disease and LV dysfunction (LVEF < 50%, mean LVEF was 26 ± 12%) scheduled to undergo either revascularization or medical therapy ± implantable cardiac defibrillator (ICD) placement were recruited consecutively for CMR. All patients underwent diagnostic coronary angiography before CMR examination. After CMR examination and assessment of total myocardial scar volume, all the patients were followed for the presence of cardiovascular events, as described below. 35 patients underwent revascularization (26 patients coronary artery bypass surgery (CABG) and 9 percutaneous coronary intervention (PCI) patients) ± ICD placement and 51 patients received medical therapy ± ICD placement. All patients had previous MI. But patients with acute infarction (within seven days), unstable angina pectoris, asthma, pulmonary disease, severe valvular disease, or contraindications to the CMR examination were excluded. The study protocol was approved by the human subjects committee at Stanford university.

### Imaging protocols

All images were acquired on a 1.5-Tesla whole-body scanner (Signa, GE Healthcare, Milwaukee, WI) with the patient in a supine position using an 8-element phased-array radiofrequency coil with breath-holding and cardiac gating. Cine images of the LV in short and long axes were acquired using a steady-state free precession sequence (SSFP, TR 3.8, TE 1.6, FA 45°, slice thickness 10 mm, slice gap 0). Late gadolinium enhancement (LGE) images (segmented k-space inversion recovery sequence, TR 7.1, TE 3.1, TI 200–250, slice thickness 10 mm, slice gap 0) were acquired throughout the entire LV starting at 20 min, following administration of 0.2 mmol/kg of gadolinium diethytriaminepentaacetic acid (Gd-DTPA, Magnevist^®^, Schering AG, Germany). The inversion time set to null the signal of normal myocardium after Gd-DTPA administration was adjusted during the course of the scan as necessary.

### Image analysis

Images were analyzed using MASS analysis software (MASS Analysis Plus Version 5.1, Leiden University). Automatic tracing with manual adjustment of endocardial and epicardial borders from short-axis images was performed to calculate left ventricular end-diastolic volume (LVEDV), left ventricular end-systolic volume (LVESV), and LVEF.

### Assessment of myocardial infarction by Late Gadolinium Enhancement

**Figure 1:**
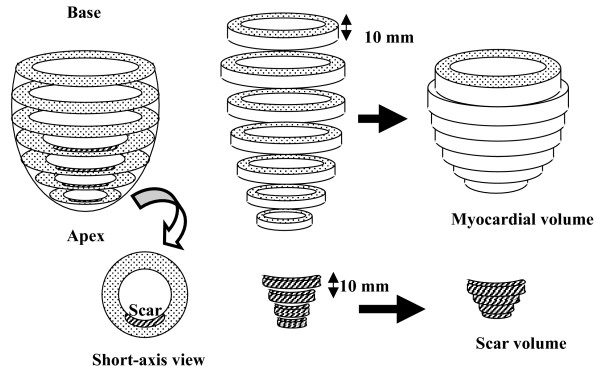
**Cardiac magnetic resonance characterization of myocardial infarction**. The total myocardial and scar area in each of the 8 to 11 short-axis images were traced manually. Myocardial and scar volume for each slice were calculated as (area myocardium or area scar × slice thickness of 10 mm). The scar percentage of myocardium was also expressed as a percentage of the total myocardial volume (Volume scar/Volume myocardium × 100).

The short-axis LGE images were evaluated for the presence of scar and traced manually to measure total scar volume. An infarct region was defined as an area of hyperenhancement, with a higher signal intensity (≥ 2 SD) compare to remote region in the same slice [13]. Myocardial and scar volume were calculated (myocardial or scar area × slice thickness of 10 mm). The scar percentage of myocardial volume was also expressed as percentage of the total myocardial volume (scar volume/myocardial volume × 100) as shown in figure 1. We analyzed contrast-enhanced images using the 72-segment model in which the left ventricle was divided into 12 circumferential segments in six short-axis views [18]. In patients with microvascular obstruction, these hypointense areas were included as scar area. Isolated midwall or subepicardial hyperenhancement was excluded because this was not considered as scar area [19,20]. The transmural extent of hyperenhancement was graded as a percentage of scar tissue in each segment: 0%, 1 to 25%, 26 to 50%, and 51 to 75% (non-transmural); and 76 to 100% (transmural).

## Results

### Patient characteristics

Clinical characteristics of the study population are summarized in table 1. Thirty-five patients were revascularized (26 CABG, 9 PCI patients). Clinical follow-up over a period of 20 ± 16 months (minimum period was 1 month, maximum period was 56 months) was obtained in all patients. Twenty-four of the 86 patients (28%) had 2 vessel disease (left main, or two vessel with proximal left anterior descending artery), and 49 patients (57%) had 3 vessel disease. During the follow-up period, 33 patients (38%) had cardiovascular events (mean follow-up period was 20 ± 16 months). Fifteen patients of the 33 patients had CHF, 9 patients had ventricular arrhythmia, 2 patients had syncope, 2 patients had MI, 5 patients needed revascularization and 6 patients died due to cardiovascular cause during the follow-up period. There were any deaths or events were related to revascularization procedures.

**Table 1:** Clinical profile of patients

	All (n = 86)	Revascularization (n = 35)	No Revascularization (n = 51)	*p*-value
Age, years	57 ± 12	58 ± 11	57 ± 14	0.32
Gender, male	73 (85%)	32 (91%)	41 (80%)	0.16
History of hypertension	54 (63%)	26 (74%)	28 (55%)	0.07
History of HL	59 (69%)	25 (71%)	34 (67%)	0.64
History of diabetes	24 (28%)	11 (31%)	13 (25%)	0.55
History of smoking	26 (30%)	12 (34%)	14 (27%)	0.50
Beta Blocker Use	62 (78%)	27 (90%)	35 (71%)	0.06
Ace-I or ARB Use	45 (57%)	17 (57%)	28 (57%)	0.36
Coronary anatomy, n (%)				
1 vessel disease	13 (15%)	3 (9%)	10 (20%)	0.16
2 vessels disease†	24 (28%)	7 (21%)	17 (33%)	0.17
3 vessels disease	49 (57%)	25 (71%)	24 (47%)	0.02
ICD placement (%)	31 (36%)	8 (23%)	24 (47%)	0.02
Scar volume (cm^3^)	13.7 ± 12.7	14.5 ± 12.0	13.1 ± 13.3	0.60
Scar % of myocardium (%)	8.3 ± 6.9	9.2 ± 7.6	7.8 ± 6.3	0.37
LVEF (%)	26 ± 12	29 ± 13	24 ± 11	0.10
LVEDV (ml%)	231 ± 83	230 ± 97	232 ± 73	0.88
LVESV (ml%)	177 ± 83	173 ± 96	180 ± 74	0.69
LVED mass/volume (g/ml)	0.79 ± 0.30	0.83 ± 0.28	0.77 ± 0.31	0.22
Ant, sep infarct lesion	55 (64%)	23 (66%)	32 (63%)	0.78
Lat infarct lesion	40 (47%)	16 (46%)	24 (47%)	0.53
Inf infarct lesion	63 (73%)	25 (71%)	38 (75%)	0.75
Cardiovascular events	33 (38%)	16 (46%)	17 (33%)	0.24

Values are expressed as a mean ± SD. HL = hypercholesterolemia;

Ace-I = Angiotensin Converting Enzyme – Inhibitor, ARB = Angiotensin Receptor Blocker ICD = implantable cardiac defibrillator; LVEF = left ventricular ejection fraction;

LVEDV = left ventricular end diastolic volume; LVESV = left ventricular end systolic volume;

† 2 vessel disease include P-LAD or LMT.

### Cardiac magnetic resonance parameters to predict cardiovascular events

CMR parameters among the patients ± cardiovascular events are summarized in table 2. Both mean scar volume and scar percentage of the myocardium were larger in patients with cardiovascular events than those without cardiovascular events (16.8 ± 12.4 cm3 vs. 11.7 ± 12.6 cm3, p = 0.023 and 10.2 ± 6.9% vs. 7.2 ± 6.7%, p = 0.037, respectively). There were no significant differences in LVEDV, LVESV and LVEF between the patients with and without cardiovascular events (234 ± 76 ml vs. 230 ± 88 ml, 180 ± 73 ml vs. 175 ± 90 ml, 25 ± 10% vs. 27 ± 13%, respectively).

**Table 2:** Predictors of cardiovascular events

	Cardiovascular events (+)	Cardiovascular events (-)	*p *– value
Scar volume (cm^3^)	16.8 ± 12.4	11.7 ± 12.6	0.023
Scar % of the myocardium (%)	10.2 ± 6.9	7.2 ± 6.7	0.037
LVEF (%)	25 ± 10	27 ± 13	0.26
LVEDV (ml)	234 ± 76	230 ± 88	0.41
LVESV (ml)	180 ± 73	175 ± 90	0.40
LVED mass/volume (g/ml)	0.73 ± 0.25	0.84 ± 0.32	0.06

Values are expressed as a mean ± SD. LVEF = left ventricular ejection fraction; LVEDV = left ventricular end diastolic volume; LVESV = left ventricular end systolic volume.

The transmurality of the scar was also analyzed by dividing into non-transmural vs. transmural extent of MI (1–75% vs. 76–100%, respectively) among the patients as shown in table 3. Patients with cardiovascular events had larger number of non-transmural segments than patients without cardiovascular events (18.4 ± 14.0% vs. 13.8 ± 11.2%, p = 0.049). Specifically, higher incidences of cardiovascular events were seen in the 26–50% non-transmural segments (9.2 ± 10.6% vs. 3.2 ± 3.6%, p = 0.03). In subanalysis, there were no significant difference in the number of both non-transmural segments and transmural segment in patients with and without ventricular arrhythmia (p = 0.71, p = 0.64, respectively). In addition, there were no significant difference in the number of both non-transmural segment and transmural segment in patients with and without worsening CHF (p = 0.94, p = 0.06, respectively).

**Table 3:** The proportion of non-transmural vs. transmural segments of myocardial infarction

	Cardiovascular events (+)	Cardiovascular events (-)	*p *– value
Non-transmural MI (1–75% scar of myocardium)	18.4 ± 14.0	13.8 ± 11.2	0.049
1 – 25% scar of myocardium	9.2 ± 11.0	6.7 ± 9.3	0.12
26 – 50% scar of myocardium	9.2 ± 10.6	3.2 ± 3.6	0.03
51 – 75% scar of myocardium	3.5 ± 4.2	4.0 ± 4.5	0.30
Transmural MI (76–100% scar of myocardium)	5.8 ± 10.2	7.2 ± 11.4	0.28

Values are expressed as a mean ± SD.

### Correlation between scar characteristics and functional parameters

**Figure 2:**
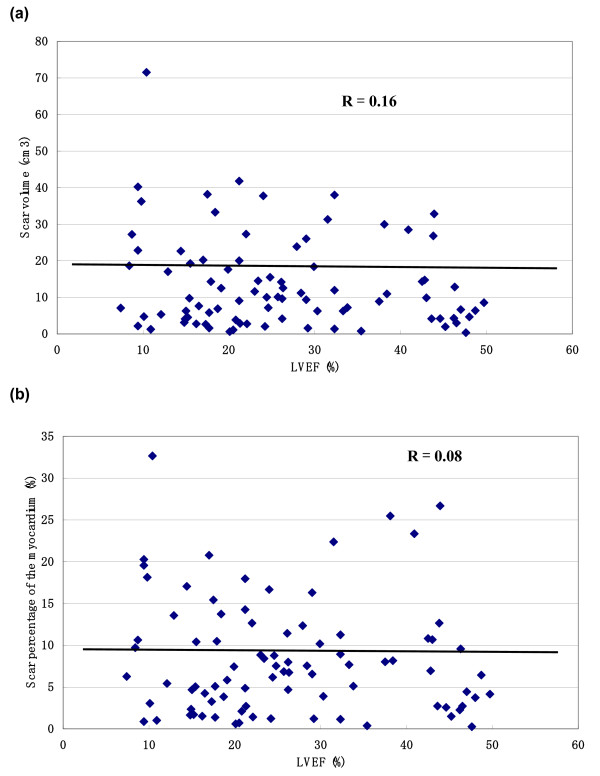
**The correlation between left ventricular volumes and the scar volume and scar percentage**. The correlation of LVEF with scar volume (a) and scar percentage of the myocardium (b).

**Figure 3:**
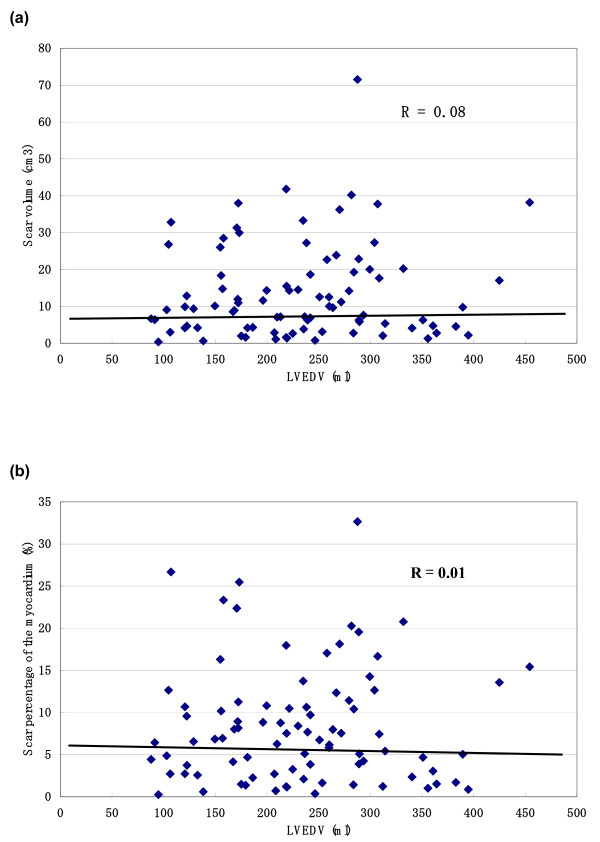
**The correlation between left ventricular volumes and the scar volume and scar percentage**. The correlation of LVEDV with scar volume (a) and scar percentage of the myocardium (b).

**Figure 4:**
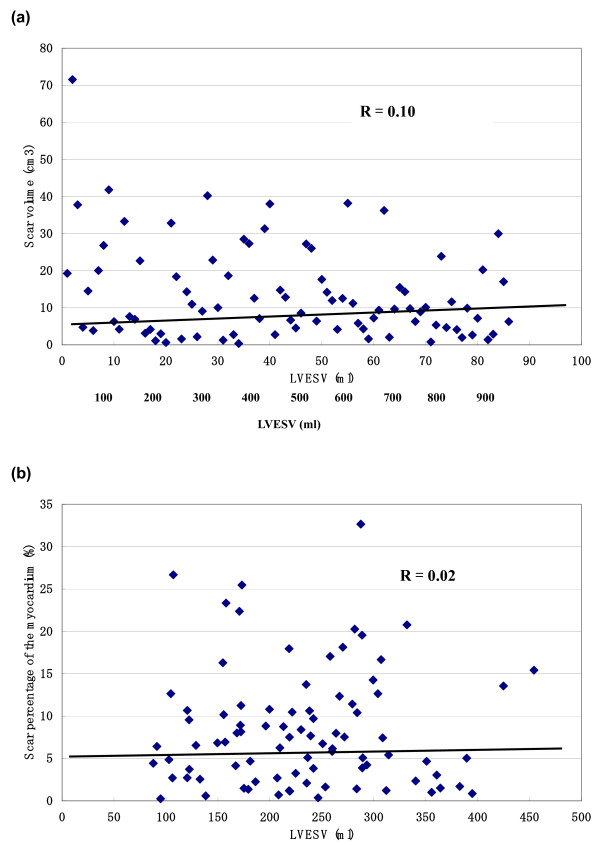
**The correlation between left ventricular volumes and the scar volume and scar percentage**. The correlation of LVESV with scar volume (a) and scar percentage of the myocardium (b).

The correlation of LVEF, LVEDV and LVESV with scar volume and scar percentage of the myocardium is shown in figure 2, 3, 4 (figure 2: LVEF, figure 3: LVEDV, figure 4: LVESV). These measurements did not correlate with scar volume with r values of 0.16, 0.08 and 0.10, respectively and did not correlate with scar percentage of the myocardium with r values of 0.08, 0.01 and 0.02, respectively.

### Effects of revascularization

When the patients were analyzed between the revascularization (CABG or PCI ± ICD placement) and the no revascularization groups (medical therapy ± ICD placement), there were more patients with 3 vessel disease and with ICD placement who underwent revascularization (p = 0.02, for each) as shown in table 1. However, there was no significant difference in both scar volume and percentage of the myocardium between the 2 groups (p = 0.60 and 0.37, respectively). Finally, there was no significant difference in the incidence of cardiovascular events between these two groups (p = 0.24).

### Clinical parameters to predict cardiovascular events

Analyses of clinical characteristics of all patients with and without cardiovascular events are summarized in table 4. There was no significant difference in clinical parameters between these two groups.

**Table 4:** Predictors of cardiovascular events

	Cardiovascular events (+) (n = 33)	Cardiovascular events (-) (n = 53)	*p*-value
Age, years	56 ± 13	58 ± 12	0.18
Gender, male	28 (85%)	45 (85%)	0.99
History of hypertension	22 (67%)	32 (60%)	0.56
History of hyperlipidemia	22 (67%)	37 (70%)	0.76
History of diabetes	11 (33%)	13 (25%)	0.38
Beta Blocker Use	25 (81%)	37 (77%)	0.10
Ace-I or ARB Use	18 (58%)	27 (56%)	0.25
History of smoking	13 (39%)	13 (25%)	0.14
Coronary anatomy, n (%)			
1 vessel disease	3 (9%)	10 (19%)	0.22
2 vessels disease	10 (30%)	14 (26%)	0.70
3 vessels disease	20 (61%)	29 (55%)	0.59

Values are expressed as a mean ± SD. Ace-I = Angiotensin Converting Enzyme – Inhibitor, ARB = Angiotensin Receptor Blocker

## Discussion

Patients surviving MI and developing chronic ICM are at risk for developing cardiovascular events. However, not all patients with chronic ICM have similar risk profiles. Traditional clinical indicators (LVEF, NYHA functional class, and coronary anatomy) and electrocardiographic markers (QRS duration, T-wave alternans, and signal average ECG) have been used to identify patients at risk for developing ventricular arrhythmia and sudden death [21,22]. However, these markers have low predictive value. They are descriptors of myocardial and electrical dysfunctions rather than specific physiologic markers to identify patients at risk for developing cardiovascular events[21]. This is the first study to confirm that the quantitation of the scar volume and percentage by CMR can predict the development of cardiovascular events in patients with ICM. The traditional risk factors including LVEF, LVEDV, and LVESV did not differentiate the patients who did and did not develop cardiovascular events. Furthermore, these parameters did not correlate with the quantitative characterizations of myocardial infarction by CMR.

Based on our study, a more detailed analysis of the injured myocardium using CMR may predict the future occurrence of cardiovascular events in patients with ICM. We have performed an extensive analysis of image-based data using CMR to identify specific tissue characteristics of the infarcted myocardium that may serve as a longitudinal prognostic marker for developing cardiovascular events in patients with ICM. In addition to the scar volume and percentage, patients with non-transmural segments had developed more cardiovascular events than patients with transmural segments. Recent studies indicate that injured but viable myocytes in the peri-infarct territory consistent with non-transmural infarction may lead to cardiovascular events [23,24]. The presence of residual viable myocardium, as a path of conduction and/or site of peri-infarct ischemia, may be a necessary cofactor in the pathogenesis of cardiovascular events, a conclusion that has been reported in previous studies [23,24]. Ejection fraction is good predictor of cardiovascular events, but it could not reveal all of LV remodeling. Therefore, a more precise predictor may be possible when both infarct size and presence of peri-infarct injury can be considered through a more quantitative evaluation of the non-transmural extent of MI.

## Conclusion

Quantitative characterization of the scar volume and percentage by CMR is superior to LVEDV, LVESV, and LVEF in prognosticating the future likelihood of the development of cardiovascular events in patients with ICM. This patient population could benefit from this study in which the scar volume and percentage measured by CMR has been validated as a quantitative prognostic marker.

## Abbreviations

CHF: congestive heart failure, ICM: ischemic cardiomyopathy, LV: left ventricular, CMR: cardiac magnetic resonance, LVEF: left ventricular ejection fraction, MI: myocardial infarction, ICD: implantable cardiac defibrillator, CABG: coronary artery bypass graft surgery, PCI: percutaneous coronary intervention, SSFP: steady-state free precession sequence, LGE: late gadolinium enhancement, Gd-DTPA: gadolinium diethytriaminepentaacetic acid, LVEDV: left ventricular end diastolic volume, LVESV: left ventricular end systolic volume, NYHA: New York Heart Association.

## Competing interests

The author(s) declare that they have no competing interests.

## Authors' contributions

HY, CKK, and PN contributed to the data collection, data analysis, and manuscript preparation. JMP contributed to the MRI sequence, data analysis, and manuscript review. MVMcC and PCY contributed to the study design, data interpretation, and manuscript review. All authors approved the final version of the manuscript submitted. Finally, JMP, MVMcC, and PCY receive research support from GE Healthcare, Inc. All research support is acknowledged on the title page of the manuscript.

## References

[B1] American heart association (2001). 2002 Heart and Stroke Statistical Update.

[B2] Emond M, Mock MB, Davis KB (1994). Long-term survival of medically treated patients in the Coronary Artery Surgery Study (CASS) Registry. Circulation.

[B3] Wong M, Staszewsky L, Latini R (2004). Severity of left ventricular remodeling defines outcomes and response to therapy in heart failure: Valsartan heart failure trial (Val-HeFT) echocardiographic data. J Am Coll Cardiol.

[B4] Poole-Wilson PA, Uretsky BF, Thygesen K (2003). Mode of death in heart failure: findings from the ATLAS trial. Heart.

[B5] St John Sutton M, Lee D, Rouleau JL (2003). Left ventricular remodeling and ventricular arrhythmias after myocardial infarction. Circulation.

[B6] Brugada J, Aguinaga L, Mont L (2001). Coronary artery revascularization in patients with sustained ventricular arrhythmias in the chronic phase of a myocardial infarction. J Am Coll Cardiol.

[B7] Every NR, Fahrenbruch CE, Hallstrom AP (1992). Influence of coronary bypass surgery on subsequent outcome of patients resuscitated from out of hospital cardiac arrest. J Am Coll Cardiol.

[B8] Kelly P, Ruskin JN, Vlahakes GJ (1990). Surgical coronary revascularization in survivors of prehospital cardiac arrest: its effect on inducible ventricular arrhythmias and long-term survival. J Am Coll Cardiol.

[B9] Manolis AS, Rastegar H, Estes NA (1993). Effects of coronary artery bypass grafting on ventricular arrhythmias. Pacing Clin Electrophysiol.

[B10] Puljevic D, Smalcelj A, Durakovic Z (1998). Effects of postmyocardial infarction scar size, cardiac function, and severity of coronary artery disease on QT interval dispersion as a risk factor for complex ventricular arrhythmia. Pacing Clin Electrophysiol.

[B11] De Sutter DJ, Tavernier R, Van de Wiele C (2000). Infarct size and recurrence of ventricular arrhythmias after defibrillator implantation. Eur J Nucl Med.

[B12] Paganelli F, Barnay P, Imbert-Joscht I (2001). Influence of residual myocardial ischaemia on induced ventricular arrhythmias following a first acute myocardial infarction. European Heart Journal.

[B13] Bello D, Fieno DS, Kim RJ (2005). Infarct morphology identifies patients with substrate for sustained ventricular tachycardia. J Am Coll Cardiol.

[B14] Bolick D, Hackel D, Reimer K, Ideker R (1986). Quantitative analysis of myocardial infarct structure in patients with ventricular tachycardia. Circulation.

[B15] Jones-Collins B, Patterson R (1981). Quantitative measurement of electrical instability as a function of myocardial infarct size in the dog. Am J Cardiol.

[B16] Karagueuzian H, Fenoglio J, Weiss M, Wit AL (1979). Protracted ventricular tachycardia induced by premature stimulation of the canine heart after coronary artery occlusion and reperfusion. Circ Res.

[B17] Wilber DJ, Lynch JJ, Montgomery D (1985). Postinfarction sudden death: significance of inducible ventricular tachycardia and infarct size in a conscious canine model. Am Heart J.

[B18] Kim R, Wu E, Rafael A (2000). The use of contrast-enhanced magnetic resonance imaging to identify reversible myocardial dysfunction. N Engl J Med.

[B19] Wu KC, Zerhouni EA, Judd RM (1998). Prognostic significance of microvascular obstruction by magnetic resonance imaging in patients with acute myocardial infarction. Circulation.

[B20] Mahrholdt H, Wagner A, Judd RM (2005). Delayed enhancement cardiovascular magnetic resonance assessment of non-ischemic cardiomyopathies. Eur heart journal.

[B21] Huikuri H, Castellanos A, Myerburg RJ (2001). Sudden death due to cardiac arrhythmia. N Engl J Med.

[B22] Santana CA, Shaw LJ, Garcia EV (2004). Incremental prognostic value of left ventricular function by myocardial ECG-gated FDG PET imaging in patients with ischemic cardiomyopathy. J Nucl Cardiol.

[B23] Tsukiji M, Nguyen P, Narayan G (2006). Peri-infarct ischemia determined by cardiac magnetic resonance evaluation of myocardial viability and stress perfusion predicts future cardiovascular events in patients with severe ischemic cardiomyopathy. J Cardiovasc Magn Reson.

[B24] Yan AT, Shayne AJ, Brown KA (2006). Characterization of the peri-infarct zone by contrast-enhanced cardiac magnetic resonance imaging is a powerful predictor of post-myocardial infarction mortality. Circulation.

